# Type of High School Predicts Academic Performance at University Better than Individual Differences

**DOI:** 10.1371/journal.pone.0163996

**Published:** 2016-10-03

**Authors:** Benjamin Banai, Višnja Perin

**Affiliations:** 1 Independent researcher, Osijek, Croatia; 2 Croatian Employment Service, Zadar Regional Office, Zadar, Croatia; University of New Mexico, UNITED STATES

## Abstract

Psychological correlates of academic performance have always been of high relevance to psychological research. The relation between psychometric intelligence and academic performance is one of the most consistent and well-established findings in psychology. It is hypothesized that intelligence puts a limit on what an individual can learn or achieve. Moreover, a growing body of literature indicates a relationship between personality traits and academic performance. This relationship helps us to better understand how an individual will learn or achieve their goals. The aim of this study is to further investigate the relationship between psychological correlates of academic performance by exploring the potentially moderating role of prior education. The participants in this study differed in the type of high school they attended. They went either to gymnasium, a general education type of high school that prepares students specifically for university studies, or to vocational school, which prepares students both for the labour market and for further studies. In this study, we used archival data of psychological testing during career guidance in the final year of high school, and information about the university graduation of those who received guidance. The psychological measures included intelligence, personality and general knowledge. The results show that gymnasium students had greater chances of performing well at university, and that this relationship exceeds the contribution of intelligence and personality traits to university graduation. Moreover, psychological measures did not interact with type of high school, which indicates that students from different school types do not profit from certain individual characteristics.

## Introduction

Since the early days of psychology, prediction of academic performance (AP) has been of high relevance to psychologists [1, 2]. This is due to the importance of AP in the life of every individual–it confines the range of possible job opportunities, as well as career choices. Higher educational level is often a prerequisite for more demanding jobs, which can also lead to greater financial outcomes [3]. Moreover, a measure of academic success can also play an important role in the job application process, as a source of information about the candidate’s prior performance [4]. Overall, it could be stated that the educational level achieved plays a role in the quality of life [5] and well-being [6] of an individual. Literature on psychological correlates of AP in post-secondary education indicates that there is a well-established relationship between intellectual abilities and AP [7, 8, 9], that individual differences in personality can explain additional variance in AP [10, 11, 8], and that general knowledge (GK) might be a valuable predictor of AP as well [12, 13]. Besides psychological individual differences, high-school success has traditionally been used in research to predict AP [14–16], mostly operationalized as grade-point average (GPA) or as a result in standardized tests (such as SAT in the USA). High-school GPAs has also been widely used as university admission criteria.

However, if a certain education system does not have standardized tests at the end of high school, GPA scores obtained by students from different high schools are hardly comparable. In Croatia this type of school system was present prior to the year 2010, when the national state exam was introduced. Until 2010, all high-school students that were enrolled in 4-year programmes were eligible university candidates. We can make a broad distinction between two types of 4-year high-school programmes: gymnasium, which offers a general education aimed to prepare students for higher education; and vocational schools, aimed to prepare students for the labour market. However, successful leavers of both types of high school are allowed to apply for admission to universities.

Therefore, in this paper, besides psychological predictors (intelligence, personality, GK), we explored the moderating role of high-school type in predicting AP.

### Intelligence

Intelligence is a general mental ability that reflects a broad capability for comprehending our surroundings, solving problems, planning, or learning from experience, while it does not reflect a set of narrow academic skills [17].

AP has been the validity criterion for psychometric measures of intellectual ability [18], since their main objective in the early days was prediction of academic success or failure [19]. Ever since, measures of intellectual abilities have become some of the most frequently-used psychological instruments, often used in employee selection [20], career guidance [21] and clinical practice [22].

Robust findings show that intellectual abilities are positive predictors of success in a variety of scholastic tasks, in level of education, and in work performance [19, 18, 23–32]. Association between intelligence and AP differs slightly with respect to different measurement methods, and varies across the level of education in different studies. Correlation between intellectual abilities and AP declines with age, being highest at primary school (.60–.70), and lowest at graduate level (.30–.40) [26, 33]. This decline is usually explained by the restriction of range in the university population, since fewer students continue education after high school [34]. Moreover, intellectual abilities are associated with continuing to higher levels of education [35].

Studies show that intelligence has high longitudinal stability in the period from childhood to early adulthood [36, 37], which makes it suitable for long-term predictions of AP at university. For example, a longitudinal study showed that psychometric intelligence measured at age 11 makes a large contribution to scores in national examinations in 25 academic subjects at age 16 [38]. Verbal and numeric aptitudes measured in 7^th^ grade were moderate-to-high predictors of total grade and grades in four academic subjects in 10^th^ grade [39]. Moreover, intellectual ability measured in the first year of university was in low correlation with academic success at the end of the first and third years of study [40].

### Personality traits

Even though some studies showed that intelligence explained more variance in academic achievement than did personality factors [41], a role for personality traits should not be excluded from the prediction of AP. It has been shown that both intelligence and personality can be related to successful learning [42]. In addition, different conceptualizations of intelligence and personality may also explain different aspects of academic performance. Whereas intelligence represents a set of specific abilities and puts a limit on *what* an individual can do, personality traits might indicate *how* an individual will do it [43]. Traditionally, academic performance has been considered to be more closely related to intellectual abilities than personality traits [44]. However, abundant literature indicates that personality traits contribute to AP as well [24, 45–49]. In this introduction, we further present relations between AP and Eysenck’s Gigantic Three personality factors: Psychoticism, Extraversion and Neuroticism (used in this study).

Neuroticism has generally been shown to negatively predict AP [19, 50] and this effect decreases with academic level [51]. It is argued that stress, impulsiveness and anxiety may influence AP (e.g. during the taking of an exam) in the same way they negatively relate to psychometric intelligence score [24], since both AP and IQ are measured with maximum-performance tests. It could also be possible that Neuroticism influences AP in other ways. Neurotic students are more often ill during examinations, which might lower their AP [52]. In addition, Neuroticism might direct a student’s attention towards anxious emotions, and away from academic homework [46].

Correlations between Extraversion and AP are ambiguous across studies. It is expected that extraverts have higher levels of energy and generally more positive attitudes towards studying, which could be reflected in a desire on their part to acquire knowledge [46]. However, extraverts benefit from this trait only at lower levels of education, where there is more interaction with teachers, and where visibility in class is appreciated. It is more likely that extraverts will favour socialization over studying, which might lower their AP at higher levels of education [53]. In line with this, it has been shown that introverts outperform extraverts in secondary and tertiary education [54]. However, findings about the relationship between Extraversion and AP are inconsistent. For example, Petrides, Chamorro-Premuzic, Frederickson and Furnham [55] reported negative correlation among a high-school sample, and Chamorro-Premuzic and Furnham [44] reported positive correlation among a university sample, while Heaven, Mak, Barry and Ciarrochi [56] reported no significant correlation among a high-school sample. Correlation size and direction vary relative to method of AP estimation [24], and relative to age and level of education [49].

Psychoticism, the last of the Gigantic Three personality traits, has been systematically negatively related to AP in previous studies [43, 49, 56–58]. Psychoticism is also negatively related to some other behaviours significant to academic excellence. For example, individuals high on Psychoticism found nothing wrong with school truancy [59], and had lower levels of responsibility and interest in studies [60], and lower involvement in coursework [44].

### General knowledge

GK represents one’s ability to acquire knowledge in general [61]. It is not a clear measure of an individual’s cognitive abilities, and it is not explicitly related to a formal education. The theoretical background of this construct may be inconclusive, since some researchers consider GK a first-order factor of crystallized intelligence [12], while others consider it a first-order factor of semantic memory [62]. GK is positively correlated with general intelligence [63], and it could indicate how an individual uses mental abilities. Someone who is intellectually bright will never acquire broad knowledge if not devoted to learning, and if not in an environment that values education. On the other hand, an individual with moderate intellectual abilities, but with high motivation for learning, and in a supportive environment, will acquire more knowledge.

Furnham and Monsen [63] showed that a measure of general cognitive abilities based on general-knowledge questions was in low-to-moderate relation with academic grades. Furthermore, Furnham, Monsen and Ahmetoglu [13] showed that a GK measure was in weak positive relation with English and Maths grades. Since it has been shown that GK relates to both intellectual abilities and personality traits [12], it can be assumed that it may serve as a predictor of AP as well.

### Academic performance

AP has been measured in numerous studies, but researchers do not agree about its definition [64]. Furnham and Chamorro-Premuzic [65] proposed that this was due to familiarity with the concept. The simplest definition would be that AP is the success of individuals in formal education (elementary, secondary or tertiary education). As with inconsistency in the definition of AP, researchers have conceptualized it differently in different studies: for example, as GPA [66], first-year examination scores [40], final-year examination scores [67], course performance [68] or standardized PISA testing scores [69]. However, researchers often investigate the relation of aptitudes or personality-trait scores to students’ scores in different tests, but rarely have information on students’ broader academic achievement, such as university graduation, which is a cumulative result of all the academic tasks that students have to fulfil prior to earning a degree.

### Type of high school

In most of the European Union countries, students may choose between general and vocational programs after finishing their primary education. In some countries, continuing to higher educational levels is limited after vocational-school graduation (e.g. in Germany vocational-school students have to take additional courses prior to higher-education admissions) [70], and in some countries they are allowed to enrol in tertiary educational programs. In Croatia, there are two types of high school–gymnasiums and vocational school–and students are selected for these schools based on their primary-school grades. The gymnasium programme offers a general education that qualifies students for university studies. Gymnasium graduates are not qualified for any profession, and it is assumed that they will continue their education at tertiary educational level. Vocational high schools offer programmes of 3 years (craftsmanship and industrial professions) and 4 years (medical, economic, agricultural professions etc.) that qualify graduates for specific professions. Four-year vocational programmes offer a mix of broad basic knowledge, as well as profession-specific knowledge, and graduates may apply for tertiary education studies (while 3-year programme graduates may not apply). About 60% of 4-year Croatian vocational school students continue to university education [71]. According to the Student Integration Model [72, 73] a key factor in successful university studies is the student’s integration within academic and social systems at the university. Individual characteristics that contribute to successful integration include the individual’s goal and commitment to achieving that goal, individual attributes regarding the importance of graduation, pre-college experience (usually GPA and academic and social attainments) and socioeconomic factors such as family background. However, if some country does not have standardized final high-school exams, the GPAs obtained can hardly be compared across different high schools. From that perspective, differences in high-school programmes (such as differences in gymnasium vs. vocational programs) can lead to a student’s integration with higher educational systems being easier or more difficult.

### Overview of present study

The aim of the present study was to further investigate predictors of AP in tertiary education using longitudinal research design. AP was operationalized as a binary variable indicating whether the participant graduated at university level or not. While psychological correlates of AP are a widely-researched topic, we wanted to examine whether type of high school moderates the relationship of individual differences in intelligence, GK and personality to AP. Since the Croatian education system allows students from both gymnasium and vocational school to enter universities, the present study investigates whether individual differences or prior education contribute more to success at university.

Intelligence and personality traits are constructs that represent qualitatively distinct individual differences, and it could be expected that there will be small or insignificant correlations between them [74, 75]. However, some studies indicate that a small negative correlation between intelligence and Neuroticism [19][76] might be found, due to mediational effects of test anxiety. Moderate correlation is expected to be found between intelligence and GK [12]. Furthermore, it can be expected that intelligence [38, 63] and GK [12, 13] will be positive predictors of AP, while Psychoticism, Extraversion and Neuroticism [49] will be negative predictors of AP. From the perspective of the Student Integration Model, we can expect that gymnasium leavers would perform better at university than those from vocational schools, merely due to the differences in primary goals between the two high-school programmes. A better scholastic background may lead to higher chances of better performance at higher educational levels.

## Method

This is an archival study, in which we have cross-referenced two archives of the Zadar Regional Office of the Croatian Employment Service (CES). Usage of the archive data was authorized by the Assistant Director of the CES. A psychologist, working as a career guidance counsellor, was in charge of data collection. No personal information about any participant was ever released outside the premises of the CES Zadar archives.

The first registry used consists of the results of psychological assessment of high-school students. The CES offers a service of career guidance to all high-school students who are university candidates (leaving gymnasium or 4-year vocational school) and who seek advice in career choice. Students can voluntarily schedule a counselling session during their final high-school semester. A psychologist gives career guidance, which consists of psychological assessment, semi-structured interview and counselling session. Typically, the psychological assessment consists of measures of intellectual abilities, general knowledge and personality. Four measures of intellectual abilities are used–the Problem Test (serving as a measure of reasoning ability) and three measures from the Multifactor Test Battery (serving as narrower measures of numeric, spatial and verbal abilities)–and a measure of general knowledge, while personality is assessed by Eysenck's Gigantic Three personality dimensions. Primarily, results of psychological assessment are used for counselling purposes; but they are also kept for a long-term psychometric evaluation of the counselling process.

Information about AP was retrieved from the CES job-seeker database, the second registry used in this study. When an individual registers as a job seeker at CES, a counsellor collects their information about formal and informal education and work experience. If a participant who enrolled for career guidance during the years 2000–2005 could be found in the job-seeker database, information on their university graduation was collected (either that they graduated, or that they did not graduate). If the information presented in the job-seeker database was inconsistent, or if there was no information for a certain participant, their test results were discarded.

The dataset is available in the S1 File. Any personal information has been removed from the dataset, and only raw scores are presented.

### Participants

The participants were final-year high-school students from Zadar County, Croatia, who enrolled in career guidance counselling at the CES, Zadar, between the years 2000 and 2005. During that period, a total of 1389 students enrolled in counselling. If the participants’ data could be matched with the job-seeker registry, the data was recorded, and 826 participants (average age of 18.07 (*SD* = .70)) were included in analyses. A total of 239 (28.9%) were men, and 578 (71.1%) were women. Of these, 538 (65.1%) had enrolled in a gymnasium programme, while 291 (34.9%) had enrolled in a 4-year vocational school programme.

### Measures

#### Problem test

The Problem Test [77] is a maximum-performance test that measures reasoning ability through problem-solving tasks. It consists of 70 tasks (mostly verbal, and some numeric), and the participant is supposed to identify the problem that lies underneath the task, and report a solution for a given problem. Cronbach alpha internal reliability has been reported to lie between .85 and .95 in various studies [78]. Problem identification and solving is considered a valid cognitive-ability measure [79].

#### Multifactor test battery (MFTB)

The MFTB is a Croatian adaptation [80] of the General Aptitude Test Battery (GATB) [81], a test widely used for purposes of professional orientation and selection. In the current study, three sub-tests were used, and all of them were maximum-performance tests. MFTB 2 is a numeric test, and the participant’s task is to perform simple maths operations as quickly as possible. It consists of 50 items. MFTB 3 is a spatial-representation test made up of 40 items, which are pictures of flat, two-dimensional objects with foldable edges. The participant’s task is to mentally ‘fold’ the planes of each object, and to select the correct three-dimensional object from among four choices. MFTB 4 is a vocabulary test. It consists of 60 items, and each of them has one pair of distractors and one pair of either synonyms or antonyms. The participant’s task is to mark the two words that are either synonyms or antonyms. Split-half reliability coefficients of the tests were: .92 (MFTB 2), .88 (MFTB 3) and .92 (MFTB 4).

#### Test of general knowledge (TOIM)

General knowledge was measured by TOIM [82]; a general knowledge test constructed for assessment of 4^th^-year high-school students. It consists of 60 questions that are constructed with the aim of reflecting not knowledge acquired in school, but rather general information from different domains such as history, art, politics, geography, popular culture, sport and so on. Cronbach alpha test reliability was reported to be .92.

#### EPQ

Personality traits were measured with EPQ [83]. The questionnaire is based on Eysenck’s biological theory of personality [84], and measures the ‘Gigantic Three’ personality dimensions: Psychoticism, Extraversion and Neuroticism. Cronbach alpha coefficients were .78 for Psychoticism, .89 for Extraversion, and .86 for Neuroticism.

#### Academic performance

Information about AP was retrieved from the CES job-seeker database. If a database entry showed that the person graduated from university, AP was coded as 1; if there was clear evidence that a person did not graduate from university, AP was coded as 0. If records were unclear as to whether the person graduated or not, their data was excluded from further analysis.

## Results

Descriptive statistics and bivariate correlations among intelligence measures (PT and three subtests of MFTB), personality measures (Eysenck's PEN personality model), GK, AP and type of high school are presented in Table 1. Prior to data analyses, assumptions for inferential statistical tests were examined. Indices of asymmetry (AI) and kurtosis (KI) indicated normal distribution of continuous variables (all AI < 3; all KI < 8) [85]. Mahalanobis distances were used to identify possible multivariate outliers. One outlier was detected, and therefore excluded from subsequent analyses.

**Table 1:** Descriptive statistics and intercorrelations for measures of intelligence, GK and personality.

		M	SD	SI	KI	Correlations								
						2.	3.	4.	5.	6.	7.	8.	9.	10.
1.	PT	40.65	10.41	-.44	-.21	.43**	.55**	.58**	.58**	-.01	-.04	-.08*	.23**	.49**
2.	MFTB2	20.29	4.29	-.06	1.74		.24**	.34**	.26**	-.11**	-.03	-.05	.21**	.29**
3.	MFTB3	19.56	6.22	-.06	-.34			.35**	.34**	.05	-.02	-.11**	.13**	.20**
4.	MFTB4	36.55	9.95	-.44	-.17				.58**	-.05	-.07*	-.06	.21**	.42**
5.	GK	25.21	11.97	.05	-.72					.04	-.13**	-.06	.24**	.51**
6.	P	4.43	2.45	.79	.68						-.00	.13**	-.12**	-.06
7.	E	15.39	3.77	-.99	.73							-.24**	-.13**	-.01
8.	N	10.96	4.83	.18	-.64								0.04	-.05
9.	AP	% (graduated)		% (did not graduate)										.31**
10.	HS(g)	47.8		17.3										
	HS(v)	14.5		20.4										

PT- Problem Test; MFTB- Multifactor Test Battery; g- general intelligence factor; GK- general knowledge; P- Psychoticism; E- Extraversion; N- Neuroticism; AP- Academic performance; HS(g)- High school- gymnasium; HS(v)- High school vocational; M-Mean; SD- Standard Deviation; SI- Skewness Index; KI- Kurtosis Index

*- p < .05

**- p < .01

Weak-to-moderate positive correlations were found among all measures of intelligence and GK, which is in line with the results of previous studies [12]. Correlations between intelligence measures and personality traits are less systematic, with most of them being non-significant. Significant correlations are negative and weak. On the other hand, all measures of intelligence and GK were in weak positive correlation with AP (coded as: 0—did not graduate; 1—graduated), indicating better test scores among students who managed to graduate. In contrast, Psychoticism and Extraversion were in weak negative correlation with AP, indicating higher values among students who did not graduate.

Furthermore, measures of intelligence and GK were also related to type of high school (coded as 0—vocational high school; 1—gymnasium). Results showed moderate positive correlations between high-school type and PT and MFTB 4, while small positive correlations were found with MFTB 2 and MFTB 3. A moderate positive correlation between high school and GK was also found. All correlations indicate higher test scores for gymnasium students.

In order to examine the relationships the relationships between high-school type, intelligence, personality and GK, on one hand, and AP, on the other, binary logistic regression was conducted. Four models were tested: in Model 1, the sole contribution of high-school type was examined; in Model 2, intelligence measures and GK were added as predictors; in Model 3, personality traits were added; and lastly we added the interaction terms between the type of high school and psychometric measures of intelligence, personality and GK in Model 4. Model fit was assessed by using two pseudo-R^2^ measures–Cox and Snell R^2^, and Nagelkerke R^2^ –and increase in fit of successive models was tested with the likelihood-ratio test [86].

Prior to the analysis, the presence of multicollinearity issues among predictors was tested for. Variance inflation factors indicated no multicollinearity (all VIF < 10). Furthermore, predictors were centred around the mean, since one of the regression models (Model 4) included interaction terms [87]. The binary logistic regression results are presented in Table 2.

**Table 2:** Results of binary logistic regression with high-school type, intelligence, personality and general knowledge as predictors, and academic performance as criterion.

	Model 1			Model 2			Model 3			Model 4		
	B	Exp(B)	p	B	Exp(B)	p	B	Exp(B)	p	B	Exp(B)	p
HS	**1.35**	**3.96**	**.00**	**.97**	**2.62**	**.00**	**1.00**	**2.72**	**.00**	**1.00**	**2.73**	**.00**
PT				.00	1.00	.97	.00	1.00	.89	-.01	1.00	.78
MFTB 2				**.06**	**1.06**	**.00**	**.06**	**1.06**	**.01**	**.10**	**1.11**	**.00**
MFTB 3				.01	1.01	.62	.01	1.01	.38	-.02	.98	.49
MFTB 4				.00	1.00	.66	.00	1.00	.82	.02	1.02	.20
GK				.02	1.02	.07	.02	1.02	.09	.01	1.01	.46
P							**-.10**	**.90**	**.00**	-.07	.93	.19
E							**-.07**	**.93**	**.00**	-.04	.96	.31
N							.03	1.03	.13	.02	1.02	.43
HSxPT										.01	1.01	.69
HSxMFTB 2										-.07	.93	.10
HSxMFTB 3										.05	1.06	.09
HSxMFTB 4										-.03	.97	.16
HSxGK										.01	1.01	.64
HSxP										-.06	.94	.37
HsxE										-.06	.95	.25
HSxN										.01	1.01	.86
%	68.1			69.1			69.9			70.2		
χ^2^	79.59, df = 1, p < .01			101.33, df = 6, p < .01			126.34, df = 9, p < .01			136.45, df = 17, p < .01		
-2LL	1013.64			991.89			966.89			956.78		
Cox & Snell R^2^	.09			.12			.14			.15		
Nagelkerke R^2^	.13			.16			.19			.21		

HS- High school type; PT- Problem test; MFTB 2- Numeric test; MFTB 3- Spatial test; MFTB 4- Verbal test; GK- General knowledge; P- Psychoticism; E- Extraversion; N- Neuroticism; %- percentage of correct predictions; χ^2^- Model chi-square, -2LL- -2 Log likelihood; B- unstandardized regression coefficient; exp(B)- The odds ratio

In Model 1, the contribution of the type of high school in predicting the AP was examined. It was found that high-school type was a significant predictor (χ^2^ = 79.59, DF = 1, p < .01; Nagelkerke R^2^ = .13), indicating that gymnasium students had greater probability of university graduation. In Model 2, we added measures of cognitive performance as predictors of AP: the four intelligence tests and GK (χ^2^ = 101.33, DF = 6, p < .01). In this model, high-school type remained significant, and MFTB 2 was the only significant predictor among the cognitive performance measures, indicating that gymnasium education and higher scores in the numeric test predicted greater probability of university graduation. In addition, the likelihood-ratio test showed better overall fit for Model 2 than Model 1 (D = 21.66, DF = 6, p < .01). Next, Psychoticism, Extraversion and Neuroticism were added as predictors in Model 3. While high-school type and MFTB 2 remained significant predictors, Psychoticism and Extraversion negatively predicted university graduation. The likelihood-ratio test showed better overall fit for Model 3 than Model 2 (D = 25.09, DF = 14, p < .05).

According to this model, students who attended gymnasium, who had a higher score in the numeric test, and who reported lower levels of Psychoticism and Extraversion, had greater probability of university graduation.

Interaction terms between type of high school and psychometric measures of intelligence, personality and GK were added in Model 4. The interaction terms were not significant; nor did they significantly increase the overall model fit (D = 10.11, DF = 25, p>.05). In the further discussion, we will interpret the results of Model 3.

## Discussion

In this study, we examined the longitudinal contributions of intelligence, personality, GK and high-school type in predicting AP, operationalized as university graduation. The results presented in Table 1 indicate that students from gymnasium high school (compared to those from vocational schools) have greater scores on all measures of intellectual abilities and in GK, while students from the different schools did not differ in personality traits. Moreover, students who managed to graduate (compared to those who did not) had greater scores on all intellectual-ability measures, and in GK. In addition, they had lower levels of Psychoticism and Extraversion. However, when all predictors of AP were introduced in the regression model (Table 2, Model 3) it was shown that only high-school type, numeric ability, Psychoticism and Extraversion were related to AP. Moreover, high-school type was shown to be the best predictor of graduation. Interpretation of exponentiated unstandardized b coefficients shows that gymnasium students have 172% greater chances of university graduation, that an increase of one point in the numeric-ability test leads to 6% higher chances of university graduation, and that an increase of one point on the Psychoticism and Extraversion scales leads to a lower probability of graduation by 11% and 7%, respectively. Interaction terms between high-school type and all psychometric measures (Model 4, Table 2) were insignificant. It seems that intelligence is a weak predictor of AP, and that gymnasium students do not profit from being ‘more intelligent’. It also seems that what students learn in high school contributes more to their success at tertiary educational level than their individual differences in intelligence, GK and personality.

Previous studies have also shown that intelligence is not a good predictor of AP at post-secondary educational level [40, 88]. In line with previous studies, introduction of intelligence measures led to a small increase (0.03) in pseudo-R^2^ indices. For example, Kappe and van der Flier [89] reported that intelligence accounted for 5% of the variance in students’ GPAs, while Farsides and Woodfield [90] reported 4% of final grade being explained by intelligence. The relation between intelligence and scholastic achievement is expected to be lower at university level than at lower levels of education [26, 33], because of the restricted range of the population at university level [34].

Furthermore, it is interesting that GK was not a significant predictor of AP. Lack of this association might be due to the construction of TOIM, the GK measure used in this study. As already described in the Method section, TOIM is constructed to reflect knowledge different from that acquired in school. The lack of association between GK and AP in this study could mean that knowledge acquired in a general academic high-school programme, as offered by gymnasiums, could be far more relevant for performance at university.

Moreover, the introduction of personality traits increased the predictive and incremental validity of AP prediction, which was in line with previous findings [11, 19, 40, 49, 68]. Psychoticism was found to be negatively related to AP. This is among the most consistent findings regarding the Gigantic Three personality traits and AP [43, 49, 60]. Individuals high on Psychoticism often show behaviour such as poor cooperation with the group, have weak organizational skills, and exhibit low achievement motivation. It is also argued that Psychoticism can serve as a proxy measure for low conscientiousness [83], a Big Five trait that usually accounts for a substantial amount of variance in AP [91, 92].

Extraversion was also shown to be negatively related to AP, which is in line with previous findings [40, 53]. While pupils may benefit from a higher Extraversion level, it seems that this trait can obstruct performance in academic tasks. Extraverts might tend to spend more time socializing than studying, and could be more easily distracted from studying, thus having lower probability of university graduation. The results did not reveal a significant relation between Neuroticism and AP in this study. In a number of studies, a negative relationship between Neuroticism and AP has been demonstrated [43, 50]. One possible explanation for the absence of this relation in the present study might be related to the method of measuring AP. AP is often measured through maximum-performance tasks (e.g. test scores). In that context, the negative emotionality that comes with high Neuroticism (e.g. test anxiety) may negatively influence performance. However, university graduation is not a maximum-performance measure, and the effects of Neuroticism may be reduced when scholastic tasks are spread over several years. Neurotic students with high levels of motivation may demonstrate several behaviours for overcoming difficulties caused by anxiety: they can learn more, make an efficient plan for attending exams, or take more than one exam for the same course and improve their performance. Researchers have usually used a narrower operationalization of AP. University graduation is a result of several (usually four or five) years of studying, during which students must accomplish various academic tasks and pass numerous exams. Many factors (both internal and external) in that process may influence one’s graduation and lower the correlation between intelligence and graduation.

It should be noted that the Big Five personality dimensions might better predict differences in AP than Eysenck’s Gigantic Three model, but they could not be used in this study, since that data was not available in the present records. For a review of research on Big Five personality traits and AP, see [8, 11, 88, 93]. Two of those traits that could be particularly important in the prediction of AP are conscientiousness (representing students who are more motivated to perform well and more persistent when faced with difficulties [11]) and openness (representing students that are more imaginative, and might better manage new learning [94]). Therefore, it might be relevant for future study to explore the relationships of the Big Five traits to AP regarding students’ high-school background.

Besides intelligence and personality measures, the results of this study revealed that high-school type is a significant predictor of AP. Unsurprisingly, the results revealed that students from gymnasium high school, compared to vocational school, have a greater chance of graduating at university. It is worth noting that gymnasium students did not benefit from having greater intelligence levels than vocational-school students. (All interactions between high-school type and intelligence were non-significant.) This may imply that the high-school programme has a bigger impact on post-secondary education than do students’ abilities. In that respect, gymnasium schools fulfil their purpose of preparing students for university studies. As previously mentioned, intelligence might put a limit on what an individual can achieve, while personality might indicate how an individual will achieve it. Here, we put emphasis on the context in which an individual is making his achievement. These findings are in line with Tinto’s Student Integration Model [72, 95]. Gymnasium leavers might be better prepared for university, since their high-school programme is designed for that purpose. That might lead to better adjustment to the new study system, and greater goal commitment, leading to more persistence and, finally, greater achievement. On the other hand, vocational school programmes are divided between general education and skills that are required in the labour market. While vocational-school students can start work immediately after leaving, it seems that they might have difficulties in adapting to university studies. Although they are eligible for university admission, it seems that the current education system fails to prepare them sufficiently for post-secondary education.

The results presented have two practical implications. First, the type of prior education can be a valuable source of information in future research on AP correlates. It could be a simple measure of prior educational context in school systems similar to that in Croatia. Moreover, future studies should focus on more detailed explanation as to why gymnasium students perform better at university level. Other contextual factors that could be considered include parental support (Do more aspirant parents send their children to better schools and provide better support?), school social groupings (Do high-scoring students at the end of elementary school regress to different means in different high schools?) and university context (Do graduates from different high schools choose different universities with different standards for awarding degrees?).

The second implication relates to equal possibilities for the higher education of those leaving different types of high school. The European Union is striving to increase inclusion in higher-education programmes [96]. Although they are eligible university candidates, it seems that students from vocational schools in Croatia perform more poorly at university level. Therefore, they might benefit from some sort of additional institutional help prior to higher education. For instance, some countries (e.g. the Czech Republic, France and the Netherlands) offer additional counselling for high-school students. On the other hand, some countries offer additional preparation programmes (e.g. the Czech Republic, Luxembourg and Iceland) prior to university entry [97].

### Limitations of the present study

There are several limitations of this study that should be addressed. First, the generalizability of the data presented is limited due to the sample used in this study. The participants in this study were final-year high-school students who decided to take career guidance counselling, and who, later in their lives, registered as job seekers with the CES. It should be noted that the entire sample showed a considerably high level of achievement motivation, which manifested itself in seeking career advice, and in actively searching for a job. However, although the sample size is reasonably large, the sample is not representative of the population of students. There are final-year high-school students that did not engage in career guidance counselling (for it was not mandatory), and there are students that did engage in career guidance counselling, but later in life did not register as job seekers (which was not mandatory, either). Not engaging in career guidance can imply both high and low scholastic motivation. For example, an excellent student who achieves high grades and knows exactly which university to attend does not need career guidance. On the other hand, a student with low grades, and without any motivation to continue studies at university level, might find career guidance unattractive. Not registering as a job seeker with the CES can also imply both a successful career and lack of motivation to look for a job. Despite some limitations regarding the unrepresentative sample, the relationships between intelligence, personality and AP presented here are in line with the literature in this field, but these findings should be confirmed on a more representative sample. The final regression model shows small pseudo-R^2^ measures. It should be taken into account that this might be due to the operationalization of AP. A continuous measure of university graduation (e.g. GPA) might serve as a better dependent variable. Moreover, on the basis of the available data, it was not possible to account for differences among universities and among different departments. It could be assumed that different departments have different standards that students must meet prior to graduation. This type of distinction at university level might provide better distinction of AP.

## Conclusion

High-school type was shown to be the best predictor of AP in this study. Students leaving gymnasium, a general academic high-school programme, had greater chances of university graduation. Of all the intelligence measures, numeric ability was the only one that was related to AP. Psychoticism and Extraversion both negatively contributed to university graduation. Problem solving, and verbal and spatial abilities, alongside Neuroticism and GK, were not related to AP.

## Supporting Information

S1 File Dataset. Psychometric measures, and information about participants’ high school type and academic performance.
(XLSX)
